# Comparative chemical analysis of volatile compounds of *Echinops ilicifolius* using hydrodistillation and headspace solid-phase microextraction and the antibacterial activities of its essential oil

**DOI:** 10.1098/rsos.171424

**Published:** 2018-02-14

**Authors:** Razieh Mohebat, Mina Zare Bidoki

**Affiliations:** Department of Chemistry, Yazd Branch, Islamic Azad University, Yazd, Iran

**Keywords:** *Echinops Ilicifolius*, compositae, hydrodistillation, HS-SPME, antibacterial activity, essential oil

## Abstract

The volatile compounds from various parts of *Echinops ilicifolius* (Compositae) such as flowers, leaves and roots obtained by hydrodistillation (HD) and headspace solid-phase microextraction (HS-SPME) methods were subsequently analysed by GC and GC/MS and compared. Thirty-seven and 20 components of the flower of *E. ilicifolius* were characterized, representing 99.7% and 100.0% of the total compositions using the HD and HS-SPME methods, respectively. The major constituents of the hydrodistilled oil were identified as linalool (58.6%), geraniol (17.4%), *n*-dodecane (10.9%) and nerol (5.4%), whereas HS-SPME extract was rich in *n*-octane (34.0%), *n*-decane (25.1%), *p*-cymene (11.1%), *γ*-terpinene (5.3%) and 1,8-cineole (5.1%). *n*-Hexadecanoic acid (32.3%), linalool (16.4%) and geraniol (8.3%) were the main components among 43 constituents identified in hydrodistilled extract of the leaf, representing 99.5% of the total components detected, whereas 16 compounds representing 99.9% of the HS-SPME method were identified, among which *n*-decane (32.6%), *p*-cymene (14.0%), *n*-octane (10.3%), limonene (9.2%), *γ*-terpinene (9.1%), 1,8-cineole (7.9%) and *α*-pinene (5.9%) were the major ones. Among 23 components comprising 91.2% of the total hydrodistilled oil detected, *n*-decane (23.1%), *n*-dodecane (14.5%), silphiperfol-4,7(14)-diene (11.1%), selin-11-en-4-a-ol (9.5%), *n*-hexadecanoic acid (7.8%) and *n*-tetradecane (5.3%) were the main constituents in the root of *E. ilicifolius*, whereas 1,8-cineole (29.0%), *n*-decane (12.6%), *n*-octane (12.6%), camphor (12.0%), *p*-cymene (9.6%) and *γ*-terpinene (5.9%) were the main components among the 20 constituents characterized in HS-SPME extract, representing 99.9% of the total components detected. The antibacterial activity of the flower, leaf and root oils of *E. ilicifolius* against six Gram-positive and Gram-negative bacteria was determined using the MIC method. The growth inhibitory zone (mm) was also measured.

## 1. Introduction

*Echinops* is one of the most important and largest genera of the Compositae family comprising more than 120 species worldwide [1]. These plants are distributed from Eastern Europe to Central Asia and from the South to the tropical mountains of Africa [2], of which about 54 species are represented in Iran [3].

Diverse species of this genus are extensively applied in Chinese and Indian folk medicine as a stimulant for milk secretion, analgesic, antityphus, to expel miasma, calm stomach ache diuretic and reduce asthma attack [4,5]. In recent years, various pharmacological effects of *Echinops* have been investigated, such as antifungal [6,7], antiprotozoal [8,9], antipyretic [10], reproductive [11], hepatoprotective [12–14], anti-cancer [15–17], antioxidant [18–20], antifeedant [21], anti-inflammatory [22–24] and antibacterial [25–29]. According to our survey, only few reports are found about their immunomodulatory effects in the literature. Tehrani *et al*. investigated the effect of the methanol extract of *Echinops ilicifolius* on peripheral blood mononuclear cells proliferation and interleukin (IL)-4 secretions [30].

Like many other representatives of the family Compositae, *Echinops* species produce essential oils and volatile components, but in spite of the large size of this genus, the composition of the volatile constituents is known only for a small number of species [9,25,31–35]; therefore, developing different techniques can be used for the extraction of volatile organic components from plants. Hydrodistillation (HD) and headspace solid-phase microextraction (HS-SPME) are common extraction methods.

Herein we wish to report the analysis of the essential oils of the flowers, leaves and roots of *Echinops ilicifolius* as native plant of Yazd through HD and results have been compared with those obtained using the HS-SPME method.

## 2. Material and methods

### 2.1. Plant material

The flowers, leaves and roots of *E. ilicifolius* were collected from Mehriz County, Province of Yazd, Iran in June 2015, during the flowering stage. Voucher specimens have been deposited at the Herbarium of Research Institute of Forests and Rangelands (TARI), Tehran, Iran.

### 2.2. Hydrodistillation method

One hundred and twenty gram portions of the air-dried samples of flowers, leaves and roots of *E. ilicifolius* were separately subjected to HD for 3** **h in a Clevenger-type apparatus. The essential oils were subsequently dried with anhydrous sodium sulfate; the corresponding oils were isolated in yields at 0.5%, 0.4% and 0.7% (w/w), respectively, and stored at 4°C in the absence of daylight until GC-MS analyses.

### 2.3. Headspace solid-phase microextraction method

Three grams of each *E. ilicifolius* air-dried and powdered sample was immediately placed into a 20 ml headspace vial, and quickly sealed with silicone rubber septa and aluminium caps for the absorption of the volatile compounds. They were transferred to the headspace. The vials were heated up to 80**°**C for 20** **min while being agitated; and then introduced directly into the GC injector.

### 2.4. Identification of the volatile components by GC and GC/MS

GC analysis was carried out on an Agilent Technoloies-7890A gas chromatograph with a split/splitless injector (280**°**C), split ratio of 1 : 50 and a flame ionization detector (290**°**C). Nitrogen was used as carrier gas (1 ml min^−1^) and the capillary column used was a DB-5 (30 m ×  0.32** **mm, film thickness 0.25 µm). The column oven temperature was kept at 60°C for 3** **min and heated to 210°C with a 3°C min^−1^ rate, then increased to 240°C with 20**°**C min^−1^ rate and the final temperature held for 8.5** **min. Relative percentage amounts were calculated from the peak area without the use of correction factors.

GC-MS analysis was performed on an Agilent Technoloies-7890A equipped with a HP-5MS capillary column (30 m ×  0.25** **mm, film thickness 0.25 µm). The column temperature was kept at 60**°**C for 3** **min and programmed to 210°C at a rate of 3°C min^−1^, heated to 240°C with 20°C min^−1^ and held for 8.5** **min. The injector and GC/MS interface line were maintained at 280°C. Helium was used as carrier gas at a flow rate of 1 ml min^−1^.The ionization voltage was 70** **eV and the ion source temperature was 230°C. The mass range (*m/z*) was 50–480. The retention indices for all the components were calculated as described by Van den Dool and Kratz [36] using *n*-alkanes as a standard. The compounds were identified by comparison retention indices (RI, DB5) with data reported in the literature and by comparison of their mass spectra with the Wiley GC/MS library, Adams library, mass finder 2.1 library data published MS [37].

### 2.5. Antibacterial assay

The antibacterial activities of the essential oils of *E. ilicifolius* were determined by measuring the growth inhibitory zones against three Gram-positive and three Gram-negative bacteria. The Gram-positive bacteria included *Enterococcus faecalis* (ATCC 29212), *Staphylococcus aureus* (ATCC 25923), *Staphylococcus epidermidis* (ATCC 12228) and Gram-negative bacteria included *Proteus mirabilis* (ATCC 43071), *Escherichia coli* (ATCC 25922) and *Pseudomonas aeruginosa* (ATCC 27853). The bacteria were obtained from the Research Center of Science and Industry, Tehran, Iran. The microorganisms (obtained from enrichment culture with a bacterial concentration 1.8 × 10^6^ of the microorganisms in 1 ml of Mueller-Hinton broth (Merck, Germany), incubated at 37°C for 24** **h were cultured on Mueller-Hinton agar (Merck, Germany, medium). After drilling, 50 µl of solutions with effective concentration in DMSO (Merck, Germany) were poured in each disc. After incubation, the inhibition zone diameter was read by a ruler [38].

In the case of MIC values less than 100 µg ml^−1^, the antimicrobial activity can be good; between 100 and 500 µg ml^−1^, moderate; from 500 to 1000 µg ml^−1^, weak; and more than 1000 µg ml^−1^, inactive [39].

### 2.6. Statistical analysis

The antibacterial tests were performed in threereplications and were presented as mean ± s.d. Data were statistically analysed using analysis of variance (ANOVA) and Duncan's multiple range test by means of SPSS (standard version 19.0, SPSS Inc., Chicago, IL, USA). There was a significant difference in the value of *p* < 0.05.

## 3. Results

The chemical analysis results obtained by HD and HS-SPME methods from *E. ilicifolius* are presented in table 1. In the hydrodistilled oils of the flower and leaf were identified 33 components representing 99.7% and 43 constituents representing 99.5%, respectively. The major components in the hydrodistilled oil from the flowers were linalool (58.6%), geraniol (17.4%), *n*-dodecane (10.9%) and nerol (5.4%), and in the leaf oil of the plant, *n*-hexadecanoic acid (32.3%), linalool (16.4%) and geraniol (8.3%) were the predominant compounds. HS-SPME analysis of flowers and leaves led to identification of nine and 16 components accounting for 100.0% and 99.9%, respectively. The main components from the dried flowers of aerial parts were *n*-octane (34.0%), *n*-decane (25.1%), *p*-cymene (11.1%), *γ*-terpinene (5.3%) and 1,8-cineole (5.1%), and the major components identified in the HS-SPME extract of leaves were *n*-decane (32.6%), *p*-cymene (14.0%), *n*-octane (10.3%), limonene (9.2%), *γ*-terpinene (9.1%), 1,8-cineole (7.9%) and *α*-pinene (5.9%). The hydrodistilled oils of the flowers and leaves were rich in regard to oxygenated monoterpenes (82.1% and 35.1%, respectively) and nonterpene hydrocarbons (17.0% and 63.1%, respectively), whereas the headspace extract contained mainly monoterpene hydrocarbons (25.0% and 44.5%, respectively) and nonterpene hydrocarbons (59.2% and 44.0%, respectively). Twenty-three components comprising 91.2% of the HD method were identified in the root. The main compounds in the essential oil of *E. ilicifolius* were *n*-decane (23.1%), *n*-dodecane (14.5%), silphiperfol-4,7(14)-diene (11.1%), selin-11-en-4-a-ol (9.5%), *n*-hexadecanoic acid (7.8%) and *n*-tetradecane (5.3%), whereas 1,8-cineole (29.0%), *n*-decane (12.6%), *n*-octane (12.6%), camphor (12.0%), *p*-cymene (9.6%) and *γ*-terpinene (5.9%) were reported as the main compounds among the 12 constituents identified in the HS-SPME extract, representing 99.8% of the total components detected. The dominant compounds in the hydrodistilled oil of the leaves were nonterpene hydrocarbons (73.7%), whereas chemical compositions of the HS-SPME method comprised mainly of oxygenated monoterpenes (47.3%), nonterpene hydrocarbons (28.1%) and monoterpene hydrocarbons (24.5%).

**Table 1.** Comparative percentage compositions of the HD and HS-SPME extract from flowers, leaves and roots of *Echinops ilicifolius*. Italics indicate the main components of each method.

		flower		leaf		root	
compounds	RI^a^	HD	HS-SPME	HD	HS-SPME	HD	HS-SPME
*n*-octane	801	—	*34.0*	—	*10.3*	—	*12.6*
(2*E*)-hexenal	849	—	—	—	1.2	—	—
*n*-hexanol	862	—	—	—	—	—	2.9
*α*-pinene	932	—	4.1	—	*5.9*	—	3.3
sabinene	972	—	—	—	1.5	—	—
*β*-pinene	976	—	—	—	1.7	—	—
1-decene	988	—	—	—	—	0.3	—
myrcene	990	0.1	0.1	0.1	2.1	—	1.6
*n*-decane	998	0.2	*25.1*	0.2	*32.6*	*23.1*	*12.6*
*α*-terpinene	1015	—	—	—	0.8	—	—
*p*-cymene	1023	—	*11.1*	—	*14.0*	0.9	*9.6*
limonene	1027	0.1	4.4	0.1	*9.2*	0.1	4.1
1,8-cineole	1030	0.1	*5.1*	—	*7.9*	—	*29.0*
(*Z*)-*β*-ocimene	1035	—	—	0.1	0.1	—	—
(*E*)-*β*-ocimene	1045	0.1	—	0.1	0.1	—	—
*γ*-terpinene	1056	—	*5.3*	—	*9.1*	0.8	*5.9*
*trans*-linalool oxide	1070	—	—	0.3	—	—	—
*cis*-linalool oxide	1086	0.1	—	0.3	—	—	—
linalool	1098	*58.6*	*10.7*	*16.4*	1.5	0.9	3.7
hotrienol	1102	—	—	0.1	—	—	—
*n*-nonanal	1103	—	—	—	—	0.2	—
*trans*-thujone	1115	—	—	—	—	—	2.6
methyl glutarate	1135	—	—	0.1	—	—	—
camphor	1143	—	—	0.1	2.0	0.6	*12.0*
(2*E*)-nonen-1-al	1164	Tr	—	—	—	—	—
terpinene-4-ol	1175	—	—	0.1	—	—	—
*p*-cymen-8-ol	1184	0.1	—	0.1	—	—	—
*α*-terpineol	1188	—	—	*6.1*	—	—	—
*n*-dodecane	1197	*10.9*	—	0.2	—	*14.5*	—
nerol	1226	*5.4*	—	2.6	—	—	—
neral	1239	—	—	0.1	—	—	—
geraniol	1252	*17.4*	—	*8.3*	—	—	—
geranial	1268	—	—	0.1	—	—	—
*n*-decanol	1279	0.1	—	—	—	0.2	—
thymol	1291	—	—	—	—	1.4	—
*n*-tridecane	1297	—	—	—	—	0.2	—
geranyl formate	1298	0.1	—	—	—	—	—
(2*E*,4*E*)-decadienal	1321	0.1	—	—	—	—	—
silphiperfol-4,7(14)-diene	1363	0.1	—	—	—	*11.1*	—
(*E*)-*β*-damascenone	1382	0.2	—	0.2	—	—	—
*n*-tetradecane	1398	0.1	—	0.1	—	*5.3*	—
(*E*)-*β*-damascone	1412	—	—	0.1	—	—	—
aromadendrene	1436	—	—	0.1	—	—	—
*α*-guaine	1442	0.1	—	—	—	3.3	—
geronyl acetone	1451	0.1	—	0.2	—	—	—
(*E*)-*β*-farnesene	1460	0.1	—	—	—	—	—
(*E*)-*β*-ionone	1483	0.1	—	0.5	—	—	—
*n*-pentadecane	1497	—	—	—	—	0.5	—
tridecanal	1511	0.2	—	—	—	—	—
*trans*-matricaria ester	1529	0.1	—	—	—	—	—
dodecanoic acid	1562	—	—	0.2	—	—	—
(3*Z*)-hexenyl benzoate	1568	0.1	—	3.3	—	1.3	—
globulol	1581	—	—	0.3	—	—	—
*n*-hexadecane	1597	0.2	—	0.3	—	2.5	—
tetradecanal	1610	—	—	0.5	—	—	—
selin-11-en-4-a-ol	1657	—	—	—	—	*9.5*	—
*n*-tetradecanol	1674	—	—	1.1	—	—	—
*n*-heptadecane	1697	—	—	0.4	—	—	—
pentadecanal	1712	0.1	—	1.9	—	—	—
tetradecanoic acid	1761	—	—	1.5	—	—	—
*n*-octadecane	1797	—	—	0.5	—	—	—
6,10,14-trimethyl-2-pentadecanone	1841	0.5	—	4.3	—	—	—
pentadecanoic acid	1857	0.2	—	1.6	—	—	—
di isobutyl phthalate	1862	—	—	1.9	—	—	—
methyl palmitate	1921	0.1	—	1.1	—	—	—
9-hexadecanoic acid	1949	—	—	—	—	1.5	—
*n*-hexadecanoic acid	1968	3.3	—	*36.2*	—	*7.8*	—
phytol	2120	0.1	—	4.9	—	—	—
linoneic acid	2132	0.6	—	0.8	—	2.5	—
oleic acid	2145	—	—	2.1	—	2.7	—
*n*-docosane	2196	Tr	—	—	—	—	—
monoterpene hydrocarbone		0.3	25.0	0.4	44.5	1.8	24.5
oxygenated monoterpenes		82.1	15.8	35.1	11.4	2.9	47.3
sesquiterpene hydrocarbones		0.2	—	0.1	—	3.3	—
oxygenated sesquiterpenes		0.1	—	0.8	—	9.5	—
other components		17.0	59.2	63.1	44.0	73.7	28.1
total		99.7	100	99.5	99.9	91.2	99.9

^a^Retention indices as determined on a DB-5 column using a homologous serious of *n*-alkanes.

Although hydrodistillation is the most popular, widespread and an effective conventional method for the extraction of essential oils from plants, it has some weaknesses. This method is a time-consuming and laborious process, and requires a large amount of sample. Also because of the presence of water and long heating time, it could be concluded that the essential oil has a higher percentage of sesquiterpenes. However, monoterpenes may be susceptible to chemical changes, and even some of the highly volatile constituents, such as *α*-pinene, evaporate during the removal of solvent by distillation. In contrast, HS-SPME is a simple, rapid method and free from wastewater. It can be used to volatile fractions from several plant samples simultaneously, which requires fewer samples.

The antimicrobial activities of flower, leaf and root oils of *E. ilicifolius* were assayed against six Gram-positive and Gram-negative bacteria and results are showed in table 2. The flower, root and leaf oils have significant activity against all Gram-positive and Gram-negative bacteria. *Staphylococcus aureus* and *S. epidermidis* were the more susceptible to flower oil than other bacteria. Leaf oil showed a stronger antibacterial effect on *S. aureus*, *E. faecalis*, *P. mirabilis*, *E. coli* and *P. aeruginosa*, and a moderate activity against *S. epidermidis*, while the antibacterial effect was higher against *E. faecalis* and *P. mirabilis* than *S. epidermidis, P. mirabilis*, *E. coli* and *P. aeruginosa* in root oil. The present study shows that there is positive correlation between the chemical content of the oil and their antibacterial activities.

**Table 2.** Antibacterial activity of flower, leaf and root oils of *Echinops ilicifolius*. IZ: inhibition zone (mm); MIC: minimum inhibitory concentration (µg ml^−1^); values were expressed as mean ± s.d. (*n* = 3), values in the same line with different superscripts (a–e) are differences as significant at *p* < 0.05 by the Duncan test using SPSS.

		flower oil			leaf oil			root oil			ofloxacin
	Gram	MIC	IZ		MIC	IZ		MIC	IZ		IZ
bacteria	+/−	(µg ml^−1^)	50 µl	100 µl	(µg ml^−1^)	50 µl	100 µl	(µg ml^−1^)	50 µl	100 µl	5 µg
*Enterococcus faecalis* (ATCC 29212)	+	500.0	13.66 ± 1.15^a^	18.33 ± 0.57^b^	62.5	20.66 ± 0.57^c^	24.66 ± 0.57^d^	31.25	20.66 ± 1.15^c^	25.00 ± 1.00^d^	18.00 ± 1.00^b^
*Staphylococcus aureus* (ATCC 25923)	+	125.0	24.00 ± 1.00^a^	28.66 ± 0.57^c^	15.62	24.33 ± 1.52^a^	26.66 ± 0.57^b^	31.25	24.00 ± 1.00^a^	28.33 ± 0.57^c^	28.66 ± 0.57^c^
*Staphylococcus epidermidis* (ATCC 12228)	+	125.0	21.00 ± 0.00^b,c^	23.66 ± 0.57^d^	125.0	18.33 ± 0.57^a^	22.00 ± 1.00^c^	125.0	21.33 ± 0.57^b,c^	21.66 ± 1.15^b,c^	20.33 ± 0.57^b^
*Proteus mirabilis* (ATCC 43071)	−	500.0	14.66 ± 0.57^a^	19.33 ± 1.52^b^	62.5	21.33 ± 0.57^c^	25.66 ± 1.15^d^	125.0	21.66 ± 0.57^c^	26 ± 1.00^d^	28.00 ± 1.00^e^
*Escherichia coli* (ATCC 25922)	−	500.0	16.66 ± 0.57^a^	22 ± 1.00^b^	62.5	24.00 ± 0.00^c^	29.33 ± 1.15^d^	125.0	22.66 ± 0.57^b,c^	28.33 ± 1.52^d^	31.33 ± 0.57^e^
*Pseudomonas aeruginosa* (ATCC 27853)	−	500.0	14.00 ± 1.00^a^	19.00 ± 1.00^b^	62.5	21.33 ± 0.57^c^	26.00 ± 1.00^e^	125.0	20.33 ± 0.57^b,c^	25.00 ± 0.00^d,e^	24.00 ± 1.00^d^

## 4. Conclusion

The current study is the first report involving an effective comparison of two different methods for extraction of oil compositions and volatile fractions from flowers, leaves and roots of *E. ilicifolius* including HD and HS-SPME combined with GC and GC-MS analysis. In all cases, the variety of volatile compounds in distillation was more than HS-SPME. Distillation extract from flowers and leaves of *E. ilicifolius* showed a higher percentage of oxygenated monoterpenes and a lower percentage of hydrocarbon monoterpenes than HS-SPME. Also, high molecular weight compounds were not identified in the headspace due to low volatility such as fatty acids and sesquiterpenes; therefore, HS-SPME is an easy, simple, inexpensive and solvent-free preparation technique to identify lighter compounds. Also, this work indicates that the essential oils of flower, leaf and root of *E. ilicifolius* have antimicrobial activities against six Gram-positive and Gram-negative bacteria. However, further studies on quantitative and qualitative extraction of volatile compounds from other plants using different methods and comparing them, and also evaluation of the antibacterial activities of the essential oils obtained from these plants are underway.

## Supplementary Material

Comparative chemical analysis of volatile compounds of Echinops ilicifolius using hydrodistillation and headspace solid-phase microextraction and the antibacterial activities of its essential oil

## Data accessibility

Datasets used in this study are available via the Dryad Repository (http://dx.doi.org/10.5061/dryad.37838) [40].
