# Supplementary material for: Comparative chemical analysis of volatile compounds of Echinops ilicifolius using hydrodistillation and headspace solid-phase microextraction and the antibacterial activities of its essential oil
Source: R Soc Open Sci. 2018 Feb 14;5(2):171424. doi: 10.1098/rsos.171424 (PMC5830750; doi:10.1098/rsos.171424)
Supplement: Comparative chemical analysis of volatile compounds of Echinops ilicifolius using hydrodistillation and headspace solid-phase microextraction and the antibacterial activities of its essential oil [file rsos171424supp1.docx]

Supporting Information

Comparative chemical analysis of volatile compounds of *Echinops ilicifolius* using hydrodistillation and headspace solid-phase microextraction and the antibacterial activities of its essential oil

Razieh Mohebat,* and Mina Zare Bidoki

Department of Chemistry, Yazd Branch, Islamic Azad University, Yazd, Iran

a)

b)

**Figure S1.** GC and GC/MS chromatograms of volatile compounds obtained from the flowers of *Echinops ilicifolius* by (a) hydrodistillation (HD) and (b) headspace solid-phase microextraction (HS-SPME) methods.

a)

b)

**Figure S2.** GC/MS chromatograms of volatile compounds obtained from the leaves of *Echinops ilicifolius* by (a) hydrodistillation (HD) and (b) headspace solid-phase microextraction (HS-SPME) methods.

a)

b)

**Figure S3.** GC/MS chromatograms of volatile compounds obtained from the roots of *Echinops ilicifolius* by (a) hydrodistillation (HD) and (b) headspace solid-phase microextraction (HS-SPME) methods.
